# Pervasive Platelet Secretion Defects in Patients with Severe Acute Respiratory Syndrome Coronavirus 2 (SARS-CoV-2)

**DOI:** 10.3390/cells12010193

**Published:** 2023-01-03

**Authors:** Johannes Kalbhenn, Jan-Steffen Pooth, Georg Trummer, David Kranzhöfer, Axel Schlagenhauf, Barbara Zieger

**Affiliations:** 1 Department of Anesthesiology and Critical Care, Medical Center, Faculty of Medicine, University of Freiburg, 79110 Freiburg im Breisgau, Germany; 2 Department of Emergency Medicine, Faculty of Medicine, Medical Center-University of Freiburg, 79098 Freiburg im Breisgau, Germany; 3 Department of Cardiovascular Surgery, Faculty of Medicine, Medical Center-University of Freiburg, 79098 Freiburg im Breisgau, Germany; 4 Department for General Pediatrics, Adolescent Medicine and Neonatology, University Medical Center of Freiburg, 79098 Freiburg im Breisgau, Germany; 5 Division of General Pediatrics, Department of Pediatrics and Adolescent Medicine, Medical University of Graz, 8036 Graz, Austria; 6 Division of Pediatric Hematology and Oncology, Department of Pediatrics and Adolescent Medicine, Faculty of Medicine, Medical Center-University of Freiburg, 79098 Freiburg im Breisgau, Germany

**Keywords:** COVID-19, platelet secretion, von Willebrand factor

## Abstract

Critically ill COVID-19 patients suffer from thromboembolic as well as bleeding events. Endothelial dysfunction, spiking of von Willebrand factor (vWF), and excessive cytokine signaling result in coagulopathy associated with substantial activation of plasmatic clotting factors. Thrombocytopenia secondary to extensive platelet activation is a frequent finding, but abnormal platelet dysfunction may also exist in patients with normal platelet counts. In this study, we performed analyses of platelet function and of von Willebrand factor in critically ill COVID-19 patients (*n* = 13). Platelet aggregometry was performed using ADP, collagen, epinephrin, and ristocetin. VWF and fibrinogen binding of platelets and CD62 and CD63 expression after thrombin stimulation were analyzed via flow cytometry. In addition, VWF antigen (VWF:Ag), collagen binding capacity (VWF:CB), and multimer analysis were performed next to routine coagulation parameters. All patients exhibited reduced platelet aggregation and decreased CD62 and CD63 expression. VWF binding of platelets was reduced in 12/13 patients. VWF:CB/VWF:Ag ratios were pathologically decreased in 2/13 patients and elevated in 2/13 patients. Critically ill COVID-19 patients exhibit platelet secretion defects independent of thrombocytopenia. Platelet exhaustion and VWF dysfunction may result in impaired primary hemostasis and should be considered when treating coagulopathy in these patients.

## 1. Introduction

SARS-CoV-2, which was first isolated in Wuhan, China [1], rapidly led to the COVID-19 pandemic with a high burden of morbidity and mortality (more than 500 million confirmed infections and more than 6 million deaths) (May 2022, Johns Hopkins University). COVID-19 shows a broad clinical spectrum ranging from asymptomatic to severe or even life-threatening courses [2]. The clinical hallmark of COVID-19 is acute respiratory distress syndrome (ARDS) [3,4]. In addition, critically ill patients suffer from thromboembolic events [5]. Viral infection can cause endothelial damage and shift the endothelium toward a prothrombotic phenotype. The substantial activation of plasmatic coagulation, platelet activation, and thromboinflammation is an important aspect of COVID-19 [6].

In contrast, COVID-19 patients can also suffer from severe bleeding events [7], which are most likely attributable to reduced platelet counts and coagulopathy. Thrombocytopenia has been described in patients with severe COVID-19 [8,9,10,11] and was associated with worse outcomes [9,11]. Although little evidence exists about the mechanism of thrombocytopenia in COVID-19, in analogy with other viral infections affecting the lung tissue, it could be attributed to decreased formation, increased destruction, and/or increased consumption of platelets [12,13]. One study reported a bone marrow aspiration in three patients with delayed-phase thrombocytopenia (beginning > 14 days after symptom onset) showing impaired megakaryocyte maturation [14]. Further, platelets in COVID-19 patients were found to show changes in morphology such as higher mean platelet volume, which could reflect an increase in immature platelets due to thrombocytopenia [11,15,16].

Platelet activation can be mediated by endothelial dysfunction either through direct viral infection or indirectly through secondary events, such as damage caused by neutrophils, leading to exposure of the subendothelium and microthrombus formation [17]. Extensive endothelial damage leads to increased release of von Willebrand factor (vWF) that contributes to the hypercoagulable state by increasing platelet adhesion and activation [18].

Platelets may be preactivated by viral infection as well. There is still controversy over whether human platelets express ACE2 to enable viral entry via this pathway [16,19,20], but viral RNA was found in platelets from COVID-19 patients or after incubation of platelets with the virus in vitro [19,20].

However, not much is known about the actual functional phenotype of platelets in critically ill COVID-19 patients. Additionally, substantial increases in vWF plasma levels have been reported, but detailed data on vWF function are scarce. The functional information on platelets and vWF of critically ill COVID-19 patients may help to understand potential impairments in primary hemostasis contributing to the bleeding risk.

Hence, in this study, we investigated platelet function, especially regarding platelet secretion, in patients with severe SARS-CoV-2 infection. Additionally, we evaluated vWF parameters in these patients.

## 2. Materials and Methods

### 2.1. Participants and Preanalytics

The study was conducted in the ICUs of the Department of Anesthesiology and Critical Care and the Department of Cardiovascular Surgery at the Medical Center of the University of Freiburg, Germany. It was approved by the local Ethics Committee (EK 336/20) and registered in the German Clinical Trials Register (DRKS No. 00006961). Informed consent was waived because treatment entirely followed departmental standard of care, and the study design was purely descriptive. The study adhered to the initiative for Strengthening the Reporting of Observational Studies in Epidemiology and was compliant with the International Society of Heart and Lung Transplantation ethics statement [21]. Critically ill patients with COVID-19 (*n* = 13) who were admitted to the ICU due to acute respiratory distress were included in the study. None of the patients were vaccinated against COVID-19. Patients were treated conservatively without circulatory support and without ECMO support.

Patients were submitted to a blood draw, and blood samples were processed within 1 h after collection to prevent cellular activation. Platelet-rich plasma (PRP) was prepared from all blood samples by differential centrifugation (200× *g*, 10 min) at room temperature.

### 2.2. Platelet Aggregation Measurements

Light transmission aggregometry of platelets was performed using the APACT 4.0 aggregometer (Labor Biomedical Technologies, Ahrensburg, Germany) and APACT software, as reported previously [22]. Briefly, blood was centrifuged without brake for 15 min at 111 g and room temperature. The supernatant was platelet-rich plasma (PRP) used for testing. The remaining blood was again centrifuged for 10 min at 2773× *g*. The resulting platelet-poor plasma (PPP) was used as blank using the agonists ADP (4 µmol/L), collagen (2 µg/mL), epinephrin (8 µmol/L), or ristocetin (1.2 mg/mL). Platelet counts were determined using standard laboratory techniques (Sysmex, Kobe, Japan). One patient sample with platelet counts < 50,000/µL was excluded from aggregation analysis.

### 2.3. Flow Cytometric Analysis of Platelet Function

PRP was diluted in autologous plasma to a concentration of 5 × 10^7^ platelets/mL. Platelets in PRP were stimulated using increasing concentrations of thrombin (0, 0.05, 0.1, 0.2, 0.5, and 1.0 U/mL) in the presence of 1.25 mM of the peptide Gly-Pro-Arg-Pro (GPRP) to prevent fibrin polymerization [23]. The reaction was stopped after 3 min by fixation with 1% formaldehyde in PBS for 30 min. After fixation, cells were washed in 500 μL PBS and incubated with fluorescein isothiocyanate (FITC)–conjugated anti-CD62 or FITC-conjugated anti-CD63. CD62 and CD63 expression levels after stimulation with increasing amounts of thrombin were determined as markers for α-granule (CD62) and dense granule (CD63) secretion. Platelet expression of CD 42 (GP Ib/IX) and CD41 (GPIIb/IIIa) was also determined. Binding of VWF was equally labeled with FITC-conjugated VWF after incubation with increasing concentrations of ristocetin (0, 0.2, 0.3, 0.5, 0.75, 1.0 mg/mL). For the fibrinogen binding assay, platelets were preincubated for 3 min at room temperature with fibrinogen-FITC and stimulated with increasing concentrations of adenosine diphosphate (ADP, 0, 0.25, 0.75, 2.0 µmol/L) prior to fixation, washing, and resuspension in 500 μL PBS. The expression of surface fluorescence was analyzed with a FACSCalibur flow cytometer (Becton Dickinson). Analyses were performed with platelets from COVID-19 patients in pairs with day controls exhibiting normal platelet secretion. Data were analyzed as linear arbitrary units.

### 2.4. Analysis of Von Willebrand Factor Function

VWF antigen (VWF:Ag; normal 0.6–1.5 U/L), VWF collagen binding capacity (VWF:CB; normal 0.6–1.5 U/L), and VWF multimers were determined, as described previously [24]. Briefly, VWF:Ag was measured in sodium citrate plasma using an in-house ELISA [25]. Collagen type I was immobilized on a microtiter plate, and VWF:CB in plasma was measured photometrically via the ELISA technique. Ratios of VWF:CB/VWF:Ag were calculated, reflecting the biological capacity of the available VWF to bind to collagen. Ratios < 0.7 were considered pathological and indicative of acquired von Willebrand syndrome. Assessment of VWF high-molecular weight (HMW) multimers was achieved by separation of VWF multimers on a sodium dodecyl sulfate–agarose gel and blotting on a polyvinylidene fluoride membrane. VWF was determined using appropriate primary and secondary antibodies and 3.30-diaminobenzidine/cobalt chloride. Standard human plasma was used as control. AVWS was diagnosed if HMW multimers were missing and if the VWF:CB/VWF:Ag-ratio was reduced.

### 2.5. Routine Laboratory Markers

The coagulation parameters aPTT, INR, fibrinogen, antithrombin, factor XIII, protein C, and protein S were determined with standard routine methods.

### 2.6. Statistics

Data are presented as mean ± SD. CD62/CD63 receptor densities as well as fibrinogen and VWF binding on platelets from COVID-19 patients and healthy controls at increasing thrombin/ADP/ristocetin concentrations were analyzed using ANOVA. Corrections for multiple comparisons were made using the Holm–Šídák method (*α* = 0.05), and multiplicity adjusted *p*-values were calculated for each comparison. Correlations between parameters were calculated using Spearman correlation analysis. GraphPad Prism 9.4 (Graphpad Software, San Diego, CA, USA) was used for performing calculations and creating figures. The datasets generated and/or analyzed during the current study are available from the corresponding author on request.

## 3. Results

### 3.1. Demographical Data and Markers of Inflammation and Coagulation

The cohort (*n* = 13) had a median age of 62 years (range: 46–82 years) and consisted of six female and seven male patients. Six of 13 patients died after being submitted to the ICU. Complete blood counts of each patient are listed in the supplement (Table S1). All patients received antithrombotic treatment with heparin. One patient received Plavix and was excluded from platelet function testing with ADP. None of the patients received aspirin. Nine of 13 patients exhibited clinical bleeding symptoms (lung or gastrointestinal hemorrhage, oral or vaginal bleeding, and epistaxis). Of those nine patients, three patients showed also signs of thrombosis (pulmonary embolism, colonic ischemia, finger or toe necrosis). One patient had a pulmonary embolism and no bleeding symptoms. Patients showed various abnormalities in routine coagulation parameters (Table 1), i.e., all patients had signs of coagulopathy. D-dimers were increased in all patients. Fibrinogen levels were pathologically high in 9/13 patients, and 6/13 patients exhibited thrombocytopenia. Antithrombin, factor XIII, protein C, or protein S levels were within the normal range in all patients.

### 3.2. Impaired Platelet Aggregation

**Figure 1:**
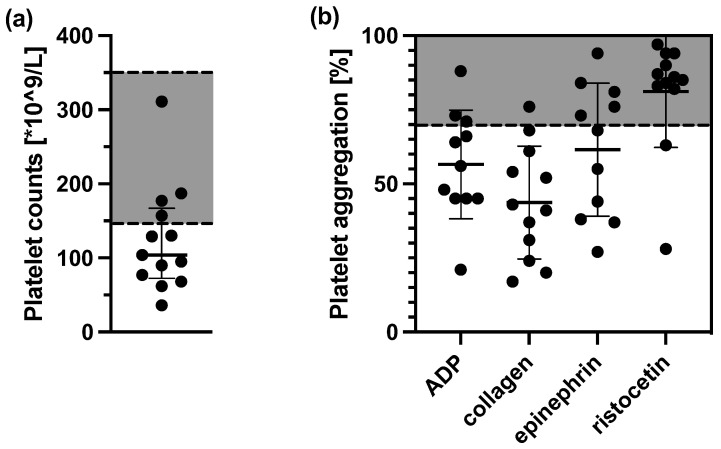
Platelet counts (**a**) and platelet aggregation analysis of COVID-19 patients (*n* = 13) after stimulation with ADP, collagen, epinephrin, or ristocetin. (**b**) Gray area marks normal range. Bars depict mean ± SD.

Platelet counts were below the normal range in 9/13 patients (Figure 1a). Platelets from COVID-19 patients showed varying hypoaggregability after stimulation with ADP, collagen, or epinephrin (Figure 1b). Eleven of 12 patients (92%) exhibited impaired aggregability with at least one agonist. Aggregation with collagen was pathological in 92%, with ADP in 72%, and with epinephrin in 55% of all cases. The degree of impairment correlated consistently between the three agonists showing general signs of hypoaggregability. In contrast, ristocetin agglutination was impaired only in 17% of all cases.

### 3.3. Platelet Secretion Defects

**Figure 2:**
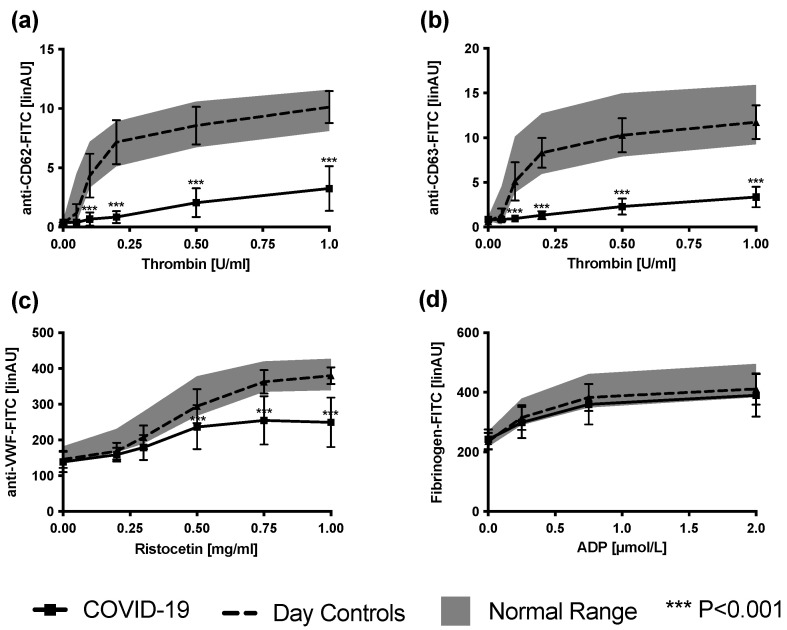
Surface expression of CD62 (**a**) and CD63 (**b**) in diluted platelet-rich plasma obtained from COVID-19 patients (*n* = 13) in comparison to day controls after stimulation with increasing concentrations of thrombin. (**c**) Von Willebrand binding after stimulation with increasing concentrations of ristocetin. (**d**) Fibrinogen binding after stimulation with increasing concentrations of ADP. Results are presented as mean ± SD. Gray area marks normal range; for difference from day controls.

Flow cytometric analysis of platelets revealed platelet secretion defects in all COVID-19 patients. CD62 and CD63 were markedly decreased after stimulation with thrombin concentrations ranging from 0.1 to 1 U/mL (Figure 2a,b).

Binding of VWF was impaired in 12 of 13 COVID-19 patients after incubation with higher ristocetin concentrations (0.5–1 mg/mL; Figure 2c). Fibrinogen binding after stimulation with ADP was unaffected in samples from COVID-19 patients (*p* = 0.201; Figure 2d). Expression of the platelet receptors GP Ib/IX (CD42) and GPIIb/IIIa (CD41) was normal.

### 3.4. Substantially Increased Levels of VWF

**Figure 3:**
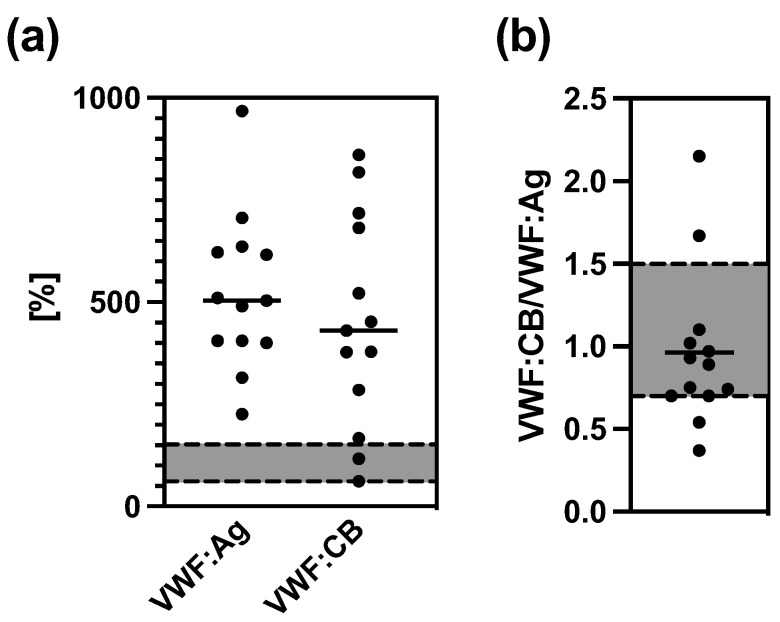
VWF:Ag and VWF:CB (**a**), and VWF:CB/VWF:Ag ratio (**b**) in COVID-19 patients (*n* = 13). Gray area marks normal range.

In all COVID-19 patients, VWF:Ag was substantially higher than the normal range (474 ± 144%, mean ± SD). Equivalently, VWF:CB was elevated in 85% of all patients (432 ± 264%, mean ± SD), pointing to extensive endothelial perturbation in this cohort. Although there was a tendency towards lower VWF:CB in relation to VWF:Ag (Figure 3a) in most patients, the VWF:CB/VWF:Ag ratio was pathologically low in only two patients, and two patients had borderline values. On the other hand, the VWF:CB/VWF:Ag ratio was pathologically high in another two patients (Figure 3b).

### 3.5. Correlations

**Figure 4:**
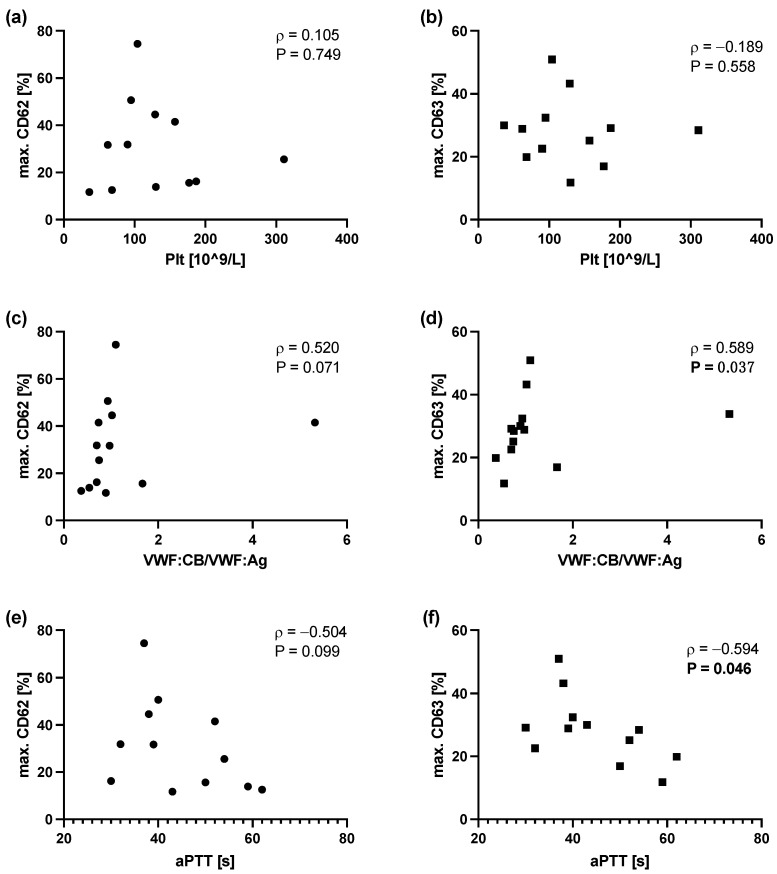
Spearman correlation analyses between maximum CD62/CD63 expression after incubation with 1 U/mL thrombin with platelet counts (**a**,**b**), VWF:CB/VWF:Ag ratio (**c**,**d**), and aPTT (**e**,**f**).

The degree of the platelet secretion defect, reflected by maximum CD62 or CD63 expression after incubation with 1 U/mL thrombin, was correlated with platelet counts, VWF:CB/VWF:Ag ratio, and the aPTT. No correlations were found between CD62 and CD63 expression and platelet count. Scatter plots reveal severe secretion defects also at normal platelet counts (Figure 4a,b). CD62 and CD63 expression did not correlate with platelet aggregation induced by ADP, collagen, or epinephrin.

CD63 expression but not CD62 expression correlated with the VWF:CB/VWF:Ag ratio (Figure 4c,d). Interestingly, when the patients with pathologically elevated VWF:CB/VWF:Ag ratios (*n* = 2) were excluded from analysis, the ratio correlated significantly with CD62 expression (*ρ* = 0.661, *p* = 0.031) and highly significantly with CD63 expression (*ρ* = 0.875, *p* < 0.001).

There was an observable tendency towards lower CD63 expression at prolonged aPTT, which was reflected in a significant inverse correlation (Figure 4e). No significant correlation was found between CD62 expression and the aPTT (Figure 4f).

VWF:Ag correlated negatively with RBC (*ρ* = −0.803, *p* < 0.001) and hemoglobin (*ρ* = −0.794, *p* < 0.001), but no significant correlations were observed between CBC parameters and the VWF:CB/VWF:Ag ratios or CD62 and CD63 expression.

## 4. Discussion

Coagulopathy and a substantially elevated risk of developing thrombosis are inherent traits of COVID-19-pathophysiology [5]. Previous studies investigating platelets from COVID-19 patients reported hyperactivity both under basal conditions and after stimulation with agonists, increased aggregation, adhesion, and spreading [16,20,26].

Platelet hyperactivation may occur due to deficient regulation of the innate immune system and substantial endothelial perturbation [17]. Additionally, platelet activation could occur through direct viral infection. Electron microscopy analysis demonstrated the presence of the virus in platelets [16,19]. It is yet unclear whether platelet activation depends on the presence of ACE2 and its interaction with the SARS-CoV-2 spike protein [16,20]. However, Koupenova et al. also showed that there exist ACE2-independent ways of platelet viral uptake [19]. It is well known that platelets are active participants in immune surveillance, expressing toll-like receptors that can recognize viruses [27]. Additionally, it has been shown that the SARS-CoV-2 spike protein engaged the CD42b receptor [28].

An alternative mechanism could be antibody-mediated platelet activation. One study showed that sera from COVID-19 patients could induce a procoagulant phenotype in platelets from healthy donors, suggesting that antibody-mediated platelet activation is central to COVID-19 dysregulated hemostasis.

Decreased platelet content of platelet factor 4 and serotonin as well as higher *p*-selectin expression argue for a degranulated state in these patients [20,26]. On the one hand, platelet hyperactivation and the release of extracellular vesicles results in a procoagulant phenotype fostering thrombosis; on the other hand, platelet depletion may lead to impaired secretion that could provoke bleeding symptoms in other tissues. However, hemostatic alterations in COVID-19 patients causing the equally occurring hemorrhagic phenotype have been insufficiently studied.

Here, we investigated potential impairments of primary hemostasis in a cohort of critically ill ICU patients with COVID-19 that were not under circulatory or ECMO support. In contrast to other COVID-19 studies measuring markers of platelet activation caused by endogenous stimuli [16,26], we analyzed the platelet function after stimulation with varying amounts of thrombin. We found a substantial reduction in alpha- and dense-granule secretion markers that was consistently observable in all patients. Our findings could explain the prevalence of bleeding symptoms, particularly mucosal hemorrhages, and are in line with another study reporting reduced ATP release in platelets from COVID-19 patients after stimulation with thrombin receptor activating peptide [29].

In contrast to other reports showing platelet hyperaggregability, we observed decreased platelet aggregation in light transmission aggregometry [20,26]. This discrepancy might be due to methodological differences (e.g., washed platelets vs. PRP) or different time points of blood draw during disease progression (before or after platelet exhaustion). While ristocetin-induced platelet agglutination was normal, flow cytometric detection of VWF binding to platelets was decreased in most patients, demonstrating higher sensitivity of flow cytometry than aggregometry to detect subtle defects in platelet adhesion. Reduced binding of VWF may be due to the aforementioned interaction of the SARS-CoV-2 spike protein with CD42b, which may competitively antagonize the platelet binding capacity of VWF [28].

Next to platelet aggregation and secretion, primary hemostasis is equally dependent on VWF functionality. In agreement with other reports, our patients showed quite high plasma levels of VWF:Ag [18,30]. While the mean VWF:CB was substantially elevated as well, the VWF:CB/VWF:Ag ratio, which indicates VWF functionality, scattered substantially across patients. Two patients showed pathologically low ratios pointing to acquired von Willebrand syndrome (AVWS). In contrast, two other patients showed pathologically elevated ratios, which were likely attributable to a quantitative imbalance between ADAMTS13 concentrations and the extensively increased VWF:Ag leading to less degradation of VWF HMW multimers and a higher risk of developing microthrombi [30]. Interestingly, VWF:Ag/VWF:CB showed highly significant correlations with platelet secretion when these patients with increased levels of VWF:CB and normal VWF:Ag/VWF:CB ratios were excluded. Hence, platelet exhaustion might indeed be more pronounced in patients with low VWF functionality, amplifying the dysfunction of primary hemostasis. The interrelation between AVWS and platelet secretion defects should be considered when COVID-19 patients suffer from bleeding symptoms (i.e., GI bleeding, mucosal bleeding). Previously, we have shown that patients under ECMO therapy rapidly develop severe AVWS and platelet secretion defects that substantially increase the risk of bleeding [31]. Interestingly, we also found a weak but significant correlation between CD63 expression and aPTT, indicating that platelet refractoriness might sometimes coincide with clotting factor consumption.

A limitation of this study is the low number of patients, potentially limiting the detection of more subtle aberrations. However, the observed dysfunctions and correlations were robust and in agreement with issues occurring in most critically ill patients with COVID-19.

## 5. Conclusions

Taking our findings together, we observed pervasive secretion defects in platelets from critically ill COVID-19 patients that are most likely attributable to platelet exhaustion. Despite elevated VWF levels, VWF function varied substantially, mirroring the clinical picture of thrombosis as well as bleeding symptoms. The observed dysfunctions in primary hemostasis should be particularly considered when patients develop bleeding symptoms.

When aforementioned platelet dysfunction is observed and clinical bleeding occurs, it seems reasonable to discontinue antiplatelet therapy if the patient is receiving antiplatelet therapy during critical illness. For treatment of bleeding, we use a step-by-step approach beginning with tranexamic acid and fibrinogen concentrate. Further treatment may include factor VIII/von Willebrand factor concentrate and transfusion of platelet concentrates [32]. In an ultima ratio situation, as rescue therapy, recombinant factor VII concentrate may be indicated in selected cases.

## Figures and Tables

**Table 1:** Routine coagulation parameters of the COVID-19 cohort.

Parameter	Mean ± SD	Pathological [*n*/Total]	Normal Range
INR	1.09 ± 0.10	4/13	0.85–1.15
aPTT [s]	45.2 ± 10.3	7/13	25.1–37.7
D-dimer [µg/mL]	5.3 ± 4.8	13/13	<0.5
Fibrinogen [mg/dL]	570 ± 203	3/10	170–420

## Data Availability

The data presented in this study are available on reasonable request from the corresponding author. Detailed methods of all procedures are provided in Section 2 of this manuscript or cited accordingly.

## Supplementary Materials

The following supporting information can be downloaded at: https://www.mdpi.com/article/10.3390/cells12010193/s1, Table S1: Complete blood counts of the COVID-19 cohort.
