# Supplementary material for: Pervasive Platelet Secretion Defects in Patients with Severe Acute Respiratory Syndrome Coronavirus 2 (SARS-CoV-2)
Source: Cells. 2023 Jan 3;12(1):193. doi: 10.3390/cells12010193 (PMC9818980; doi:10.3390/cells12010193)
Supplement: Supplementary file 1 [file cells-12-00193-s001.zip › cells-2111009-supplementary.pdf]

Table S1. Complete blood counts of the COVID-19 cohort

Patient	WBC [*10³/µl]	PLT [*10⁶/µl]	RBC [10⁶/µl]	HGB [g/dl]	HCT [%]	MCV [fl]	MCH [pg]	MCHC [g/dl]
1	8.12	95	2.55	7.7	22	86.3	30.2	35.0
2	51.5	159	2.24	6.8	21.1	94.2	30.4	32.2
3	14.57	208	2.59	7.8	25.2	97.3	30.1	31.0
4	15.17	360	2.46	7.4	21.8	88.6	30.1	33.9
5	13.52	278	3.35	9.3	28.3	84.5	27.8	32.9
6	17.32	120	2.5	7.5	22.3	89.2	30.0	33.6
7	12.38	80	2.34	7.0	19.0	81.2	29.9	36.8
8	10.20	172	2.57	7.7	23.1	89.9	30.0	33.3
9	8.52	188	2.4	7.6	24.2	100.8	31.7	31.4
10	8.49	100	2.82	8.4	23.1	81.9	29,8	36.4
11	10.4	231	4.28	12.2	35.6	83.2	28.5	34.3
12	14.54	141	3.01	9.0	27.6	91.7	29.9	32.6
13	4.98	36	2.64	7.8	23.8	90.2	29.5	32.8
Normal range	3.9-9.8	146-328	4.5-5.8	13.5-17.6	39,6-50,6	80-95.5	27.6-32.8	32.8-36.6

*WBC: White blood cell count; PLT: Platelet count; RBC: Red blood cell count; HGB: Hemoglobin; HCT: Hematocrit; MCV: Mean corpuscular volume; MCH: Mean corpuscular hemoglobin; MCHC: Mean corpuscular hemoglobin concentration
